# Are we ready to integrate advanced artificial intelligence models in clinical laboratory?

**DOI:** 10.11613/BM.2025.010501

**Published:** 2024-12-15

**Authors:** Slavica Dodig, Ivana Čepelak, Matko Dodig

**Affiliations:** 1 Department of Medical Biochemistry and Hematology, Faculty of Pharmacy and Biochemistry, University of Zagreb, Zagreb, Croatia; 2 Information System and Information Technologies Support Agency, CDU infrastructure management department, Zagreb, Croatia

**Keywords:** artificial intelligence, machine learning, deep learning, healthcare, clinical laboratory

## Abstract

The application of advanced artificial intelligence (AI) models and algorithms in clinical laboratories is a new inevitable stage of development of laboratory medicine, since in the future, diagnostic and prognostic panels specific to certain diseases will be created from a large amount of laboratory data. Thanks to machine learning (ML), it is possible to analyze a large amount of structured numerical data as well as unstructured digitized images in the field of hematology, cytology and histopathology. Numerous researches refer to the testing of ML models for the purpose of screening various diseases, detecting damage to organ systems, diagnosing malignant diseases, longitudinal monitoring of various biomarkers that would enable predicting the outcome of each patient’s treatment. The main advantages of advanced AI in the clinical laboratory are: faster diagnosis using diagnostic and prognostic algorithms, individualization of treatment plans, personalized medicine, better patient treatment outcomes, easier and more precise longitudinal monitoring of biomarkers, *etc*. Disadvantages relate to the lack of standardization, questionable quality of the entered data and their interpretability, potential over-reliance on technology, new financial investments, privacy concerns, ethical and legal aspects. Further integration of advanced AI will gradually take place on the basis of the knowledge of specialists in laboratory and clinical medicine, experts in information technology and biostatistics, as well as on the basis of evidence-based laboratory medicine. Clinical laboratories will be ready for the full and successful integration of advanced AI once a balance has been established between its potential and the resolution of existing obstacles.

## Graphical abstract

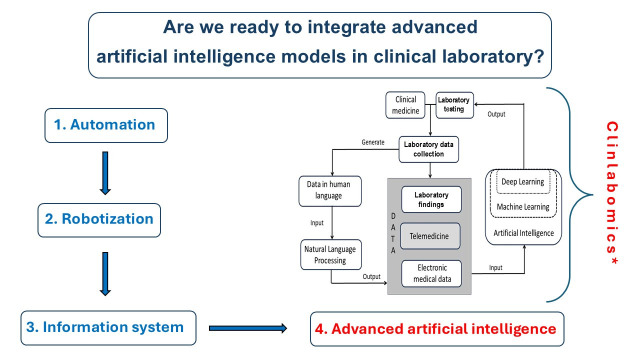

## Highlights

• Advanced artificial intelligence (AI) technology can be applied in all stages of the laboratory procedure - preanalytical, analytical and postanalytical

• Currently, there are AI algorithms for urine analysis and for the detection and monitoring of various diseases

• Before advanced AI algorithms are introduced into everyday laboratory practice, they need to be validated, standardized and regulated in ethical and legal aspects

## Introduction

The integration of artificial intelligence (AI) into numerous scientific disciplines over the past decades has opened up new opportunities in many segments of medicine and healthcare, including clinical laboratories (*1*-*3*). The development of AI in the last twenty years has led to easier management of health data, and the improvement of some diagnostic and therapeutic procedures as well as in other aspects of healthcare. The application of innovative methods not only improved the quality of healthcare, but also enabled a personalized approach to the treatment and prevention of diseases. The integration of AI in clinical laboratories brings many potential advantages, but also opens numerous questions that need to be resolved before this new technology will be fully integrated into all stages of the testing process in a routine clinical laboratory.

The aim of this review article is to give a brief overview of the structure of AI, its development especially in clinical laboratories, what are the perceptible advantages and disadvantages or problems and how they could be solved. Several examples of successful application of AI in clinical laboratories are also listed. At the end, it is indicated in which direction research into the application of AI in clinical laboratories is going. For the purpose of writing this review article, the PubMed, Medscape, ResearchGate, and National Library of Medicine were used. The literature search covered the period from 1991. A total of 98 articles were analyzed, from which 63 articles were selected. Among them, 13 references refer to the period 2005-2020, and 50 references to the period 2021-2024. Key words were „artificial intelligence“, „machine learning“, „deep learning“, „automatization“, „robotics“ „medicine and healthcare“, „telemedicine“, „laboratory medicine“, „clinical chemistry“, and „clinical laboratory“.

## Artificial intelligence

According to Encyclopedia Britannica, AI can be defined as the ability of machines (digital computer or computer-controled robot) to perform tasks mostly associated with human beings (*4*). This means that human intelligence is simulated by the use of machines that are programmed to learn and apply knowledge similar to humans. It is important that the final solution of a task is equal to or even better than the human solution. Artificial intelligence does not refer to one technology, but combines information technology (IT) and technologies that cover almost all spheres of human life (economy, engineering, healthcare, education, scientific community, *etc.*).

The basic starting point of AI is IT, which unifies computer systems (hardware, software, peripheral equipment), programming languages and data processing and storage. Two subdomains are important in the AI framework: machine learning (ML) and natural language processing (NLP) - both subdomains are complicatedly intertwined. Machine learning as one of the most common subdomains of AI, involves the development of algorithms and statistical models that enable computers to improve their performance on a task through experience. Machine learning models for analyzing data require four main components: data collection, data preprocessing, model development, and model evaluation (*5*). A specialized subdomain of ML is deep learning (DL), that involves neural networks with many layers (deep neural networks); DL has been particularly successful in tasks such as image and speech recognition (*1*, *6*). Natural language processing implies the ability of a machine to understand, interpret, and generate human-like text. Natural language processing is crucial for applications like language translation, chatbots (software designed to imitate human conversation through text or voice commands), and sentiment analysis (opinion mining; classification of texts according to positive, negative and neutral classifications). In order to be able to understand the concept of AI, it is important to understand the specific terminology of this field. Some of the most important terms are explained in Table 1.

**Table 1:** Alphabetical list of artificial intelligence terms

**Terms**	**Explanation**
Artificial intelligence (AI)	simulation of human intelligence processes by machines or computer systems
AI algorithm	programming that instructs a computer how to operate independently
Machine learning (ML)	subdomain of AI that focuses on developing algorithms and models that help machines learn from data and predict trends and behaviors, without human assistance
Deep learning (DL)	subdomain of ML that utilizes artificial neural networks for processing and analyzing a significant amount of data; it can learn from unstructured data without supervision
Natural language processing (NLP)	subdomain of AI that enables computers to understand spoken and written human language
Neural network	DL technique designed to resemble the human brain’s structure; it requires large data sets, which enables features like speech and vision recognition
Telemedicine	use of information technology to deliver medical data at a distance
Adapted from references 4 and 7.	

## Timeline of artificial intelligence in laboratory medicine

Since the first beginnings of AI (the term was established and introduced by John McCarthy in 1956 at the Dartmouth Conference), several development periods can be traced (*8*). In the first twenty years, from the 1950s to the 1970s, the focus was on the development of first machines and then robots that could follow one-step commands and more complex instructions, respectively. In the period from the 1970s to the 2000s, most of the work was done on the networking of clinical and biomedical researchers. The development of AI in medicine after the year 2000 progressed significantly.

**Figure 1:**
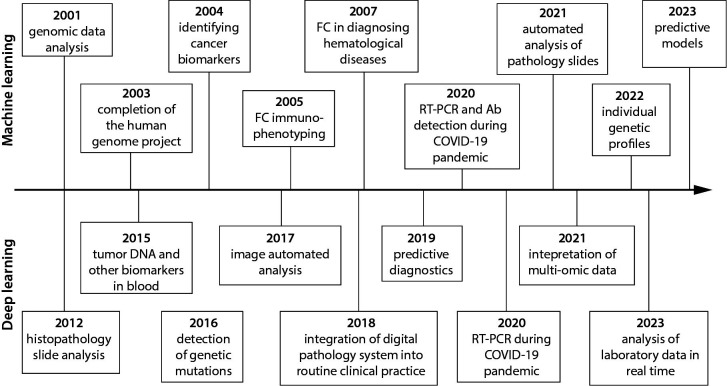
Timeline of machine learning and deep learning in clinical laboratory. FC - flow cytometry. RT-PCR - real-time polymerase chain reaction. COVID-19 - coronavirus disease caused by the SARS-CoV-2 virus. Ab - antibody.

In the clinical laboratory, the timeline for advanced AI started in the 1990s, when Artificial Neural Networks applications were used for simple tasks like pattern recognition, image analysis (*e.g.* in pathohistology), laboratory data analysis for disease progression, and for laboratory information systems (LIS). The use of ML in the recognition of pathohistological images began to become relevant in 1994. In 1997, the first automated ML algorithms were introduced to analyze large datasets, to improve the accuracy of routine diagnostic procedure. The timeline of DL was set between 1980 and 1990. It began to be applied in clinical laboratory diagnostics through automation, image analysis and precision medicine. Since the early 2000s, there has been great progress in the application of ML and then DL algorithms in clinical laboratories (Figure 1).

Artificial intelligence in laboratory medicine began to develop when laboratories were equipped with computers, analytical hardware and supporting software, which enabled the automation and robotization of a large number of analytical procedures. It was the beginning of a new era in clinical laboratory testing (*9*). The automated process of laboratory testing is unthinkable without robotic and computer technology, and that is why all three technologies are combined in analyzers of clinical laboratories. Thus, the basic parts of the automated system of the clinical laboratory are: a) analyzers; b) robotic systems that move racks with patient samples and take samples and reagents into the appropriate cuvette; and c) an information system that coordinates the entire process from patient registration and necessary analysis to entering the findings into LIS and sending the results to the hospital information system (HIS). Automated analyzers in the clinical laboratory can perform a large number of different analyses and are adapted to the type of laboratory. All analyzers are equipped with a computer, analytical hardware and supporting software and are networked in the LIS from which they are transferred to the HIS (*10*-*12*).

## Application of advanced artificial intelligence models in clinical laboratory

**Figure 2:**
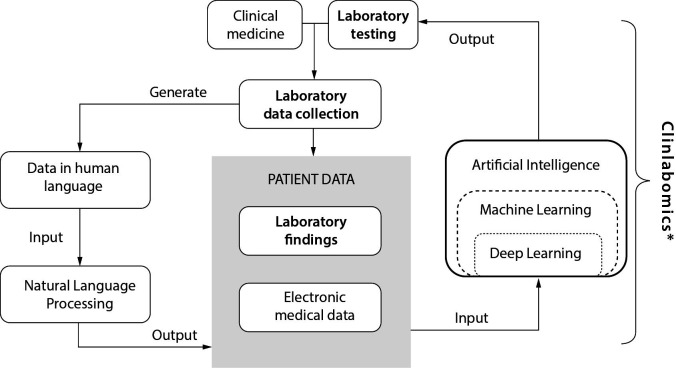
Algorithm (sequence of mathematically rigorous instructions) of artificial intelligence system in clinical laboratory. Artificial intelligence (AI) works with structured numerical data (*e.g.* values of biomarkers in serum) and unstructured digitized images (*e.g.* blood smear images). Structured data enters the neural network for analysis. Unstructured data is first brought to a form that can be analyzed using natural language processing. Natural language processing bridges human-computer communication in a way that is contextually relevant. After that, AI models (including *e.g.* machine learning and deep learning algorithms) generate clinically meaningful data, which become an integral part of each patient’s digital medical data. *In accordance with the huge amount of data that clinical laboratories generate every day in different physiological samples, and their processing with AI tools, Wen *et al.* proposed a new notion of clinical-laboratory omics within healthcare - “clinlabomics” (combines clinical laboratory data with AI) (*15*).

Despite the relatively long history of AI in clinical laboratories, the professional and scientific literature is still lacking data that would review and explain all the benefits and negativities of AI in the field of laboratory medicine. As laboratory medicine continues to undergo automation, robotization and digitalization, laboratory professionals will certainly face challenges related to the evaluation, integration and validation of AI algorithms in clinical laboratories. Integration of AI in clinical laboratories demand fulfilment of several requirements, from digital laboratory equipment, appropriate infrastructure and information technology (ML platforms, networking of LIS with HIS, digital data and medical imaging handling systems, data analysis and visualization software, advanced data storage solutions, high-performance computing systems) to trained staff. Of particular importance are solutions for patient data security and privacy. To ensure the security of patient data, advanced encryption and cyber security systems are required for data transmission and storage. In addition, the entire system must comply with regulations, such as, for example, the General Data Protection Regulation (GDPR) in Europe, especially regarding the handling of sensitive medical data (*13*, *14*). The AI implies that the results are first entered into LIS, after which they are processed by AI algorithms (Figure 2).

Machine learning and DL have an important function in the preanalytical, analytical and postanalytical phase of the testing process in the clinical laboratory (Table 2).

**Table 2:** The use of machine learning (ML) and deep learning (DL) algorithms in some automatic procedures during the preanalytical, analytical and postanalytical phases of the testing process in the laboratory and their advantages

**Phase of the analytical process (advantages)**	**Automated procedures**
**Preanalytical phase** a) *Reduction of errors*: reducing human error as routine tasks and real-time monitoring are automated b) *Efficiency and speed*: the process is accelerated and more samples can be processed in less time while maintaining accuracy c) *Optimized use of resources*: it is easier to ensure a better distribution of personnel, equipment and necessary materials, which optimizes the flow of obtaining results and reduces costs	computerized (physician) order entry calibrator sample identification (barcode size and quality) patient sample identification (barcode size and quality) determination of the HIL-indices in the patient’s serum/plasma detection of a clot in a blood sample calculation the reliability (*i.e.* accuracy and precision) of measured control samples (quality control) determination of reference intervals for individual patient populations (*e.g.* children, adults, males, females *etc.*)
**Analytical phase** a) AI-enhanced diagnostics quickly and accurately analyzes large amounts of complex laboratory data. It can detect patterns and anomalies that might be missed by manual inspection. b) ML and DL algorithms excel at recognizing patterns in data (*e.g.* biomarker profiles), which may indicate changes in a patient’s health. AI can screen through massive amounts of data and identify significant patterns faster than traditional methods.	marking results that deviate from the specific reference intervals, *e.g.* children, adults, males, females (using flags) automatic memory and processing of numerous numerical values ​​of individual biomarkers specific for various diseases (including point of care testing results) longitudinal monitoring of biomarkers in an individual patient erithrocytes and leukocytes morphology classification quantification of blood cell lineages and marking immature forms (by blood cell counter) automatic analysis of digital images, *e.g.* in hematology, cytology, histopathology (comparison with memorized images in the information system, by the use of algorithms incorporated in the software) precise detection of neoplastic cell lines and cellular markers of interest (*e.g.* flow cytometry) classification of specific molecules classification of chromosomal abnormalities
**Postanalytical phase** a) *Increased accuracy*: AI reduces the risk of human error during verification, interpretation and reporting of results. It ensures that physicians receive accurate and reliable data b) *Faster processing time*: accelerates the delivery of results to doctors and patients, especially in emergencies c) *Improved decision support*: AI provides physicians with better insights and actionable recommendations, helping them make better decisions based on comprehensive data analysis d) *Personalized care*: AI enables personalized interpretation of laboratory results, which improves quality of care	interpretation of complicated biochemical laboratory panels and molecular results (*e.g.* allergenic molecules, „omics“) use of large amounts of previous medical data to create a more personalized interpretation of obtained test results longitudinal monitoring of biomarkers and prediction the onset and behavior of diseases
The HIL-indices identifies the presence of hemolysis (H), icterus (I) and lipemia (L) in the patient’s serum/plasma. Today, the automatic spectrophotometric determination of the HIL-indices is considered the gold standard in the assessment of sample quality (*19*). AI - artificial intelligence. Adapted according to references 5, 16-18.	

The main advantages of applying AI in the clinical laboratory include: a) automation of repetitive processes; b) shortening of the time needed to obtain results; c) accurate analysis of large amounts of complex laboratory data; d) real-time monitoring and predictive diagnostics precision of results and reduced possibility of human error; f) detection of early-stage diseases by identifying patterns in laboratory test results long before clinical symptoms appear; g) improved decision support, *i.e.* help physicians make better-informed decisions; h) personalized medicine; the integration of genomics, proteomics, metabolomics, and transcriptomics data; i) easier and more precise longitudinal monitoring of biomarkers crucial for diagnosis and prediction of treatment outcomes; j) integration of laboratory data with clinical information; k) better patient treatment outcomes, *etc.*

The disadvantages relate to the: a) lack of standardization, quality of the entered data and their interpretability, since LIS’s may have incorrectly labeled or missing data; b) lack of well-established guidelines for laboratory-developed applications in digitized data; c) potential over-reliance on AI can reduce the need for human knowledge, leading to skill erosion or complacency; d) need for technology improvement and the cost of the entire IT structure of LIS and HIS (*16*, *20*).

Researchers examine a whole range of analyte/biomarker panels and their interrelationships, both laboratory and clinical, that could help in the diagnosis and prognosis of various disorders and diseases. Great attention is paid to the examination of the application of algorithms in cancers, for testing kidney diseases, liver diseases, thyroid function, for predicting the likelihood of acute myocardial infarction, *etc.* (*21*-*37*).

### Machine learning in cancer diagnosis

There are attempts to identify tumor markers that would enable the early detection of malignant processes, for example the determination of aMAP risk score (includes age, sex, platelet count, albumin and bilirubin concentration) as a predictive model for assessing the five-year risk of hepatocellular carcinoma in patients with viral and non-viral hepatitis (*23*). The aMAP risk score can be determined using ML (*24*). According to some research, there are other risk score models that could help early detection of malignant processes (Table 3).

**Table 3:** Possible artificial intelligence models for early detection of malignant diseases

**Risk score model**	**Early detection of cancers**	**Reference(s)**
Transcriptome and proteome data (machine learning)	breast cancer	(*22*)
aMAP score (machine learning): albumin, bilirubin, platelets, age, sex	hepatocellular carcinoma	(*23**,**24*)
Combination of tumor markers (neural network): CEA, AFP, CA242, CA125, CA199, CA72-4, MG7-Ag, age, sex	non-invasive gastric cancer	(*25**,**26*)
GBDT‐TC18 (machine learning): 16 routine laboratory parameters: CEA, CA19-9, CA125, AMY, glucose, total bilirubin, conjugated bilirubin, fibrinogen, RBC, Hb, Htc, WBC, LYMP%, EO-c, NEU%, P-LCR + age, sex	pancreatic ductal adenocarcinoma	(*27*)
CRC‐Lab7 (4 machine learning models): fecal occult blood, CEA, RDW, lymphocyte count, albumin/globulin ratio, HDL, HBV	colorectal carcinoma	(*28*)
Combination of conventional laboratory biomarkers (machine learning): CEA, Lp(a), HDL, Hb	colorectal carcinoma	(*29*)
Tissue and serum proteomic datasets (machine learning): HE4, CA125	high-grade serous ovarian carcinoma	(*30*)
Proteomics/panel of proteins associated with exosomes (machine learning); biomarkers for exosomes: CLTC, EZR, TLN1, CAP1, MSN	cancers	(*31*)
AFP - alpha-fetoprotein. AMY - amylase. RBC - red blood cell count. CA125 - carbohydrate antigen 125. CA19-9 - carbohydrate antigen 19-9. CA242 - carbohydrate antigen 242. CA72-4 - carbohydrate antigen 72-4. CAP1 - adenylyl cyclase-associated protein 1. CEA - carcinoembryonic antigen. CLTC - clathrin heavy chain. EO-c - absolute eosinophil count. EZR - ezrin. GBDT-TC18 - gradient boosting decision tree-based ternary classifier. Hb - hemoglobin. HBV - hepatitis B virus core antibody. HDL - high‐density lipoprotein. HE4 - human epididymis protein 4. Htc - hematocrit. Lp(a) - lipoprotein (a). LYMP% - percentage of lymphocytes. MG7-Ag - gastric carcinoma-associated MG7-Ag. MSN - moesin. NEU% - percentage of neutrophilic granulocytes. P-LCR - platelet-large cell ratio. RBC - red blood cell count. RDW - red blood cell distribution width. TLN1 - Talin-1. WBC - white blood cell count.		

Sopasakis *et al.* have shown that the gamma-globulin fraction and part of the beta-2 globulin fraction are the most important features of electrophoresis, essential for detecting the presence of M-protein, which is important for establishing the diagnosis of multiple myeloma (*38*).

### Machine learning in kidney diseases

In the urine, analytes important for diagnosing some kidney diseases are examined. Analysis of urine sediment is one of the basic tests that are used every day in the laboratory diagnosis of many diseases, *e.g.* urinary and kidney diseases, metabolic diseases, diabetes. Using urine sediment analysis, different particles such as leukocytes, erythrocytes, various types of epithelium and crystals, casts, bacteria, fungi can be detected. Sometimes problems are encountered in distinguishing some mutually similar particles in traditional individual microscopic analyses. In recent years, the application of computer-aided methods, *i.e.* DL techniques, have proven to be successful in the classification of particles in urine sediment analysis (*39*-*41*).

Modern urine sediment autoanalyzers have automated digital microscopic systems that can capture high-resolution images of the patient’s urine sediment. These microscopes and analyzers are equipped to automatically scan and digitize sample images. Once the urine sample is digitized, the DL algorithm processes the images in real time, detecting and classifying individual solid particles of urine sediment. Deep learning algorithms can analyze thousands of images in a short time.

The kidney biopsy for histopathology is considered the gold standard for the diagnosis of kidney disease. A useful ML model is the use of a filter method based on the identification and correlation analysis of key clinical predictors (age, blood pressure, hypertension, diabetes mellitus, coronary artery disease, anemia, appetite, foot edema) and laboratory predictors such as albumin, blood glucose, urea, creatinine, sodium, potassium, hemoglobin, number of leukocytes, erythrocytes, specific gravity of urine, pus cells, accumulations of pus cells and bacteria in urine (*34*). Thanks to metabolomics and ML, diabetic nephropathy can be detected by determining metabolites in serum using screening models for, for example, amino acids (tyrosine, serine, methionine) (*42*).

### Machine learning in liver diseases

Machine learning makes it possible to assess the risk of developing liver diseases. Liver diseases are mainly the result of chronic inflammation and oxidative stress (*35*). Acute inflammation stimulates the secretion of pro-inflammatory cytokines, *e.g.* interleukin-1, tumor necrosis factor alpha and prostaglandin E2. Low-grade inflammation impairs and weakens the body’s immune function. If inflammation persists, increased values of these cytokines cause low-grade inflammation that affects the development of metabolic and infectious diseases. Oxidative stress promotes the progression of liver fibrosis, and consequent liver cirrhosis or hepatocellular carcinoma (*43*).

A LiverRisk model was developed that uses two clinical parameters (age, sex) and six routine laboratory parameters: catalytic activities of aspartate aminotransferase (AST), alanine aminotransferase (ALT) and gamma-glutamyltransferase (GGT), as well as the concentration of glucose, cholesterol and number of platelets (*44*). LiverRisk score is also used for prediction of liver fibrosis, liver-related and diabetes-related mortality, which can be classified as low, medium and high risk (*45*).

### Machine learning in cardiovascular diseases

Simplified, cardiovascular diseases include angina pectoris, myocardial infarction, stroke and heart failure. Up to 90% of cardiovascular diseases can be prevented (*25*). In recent years, ML models have been developed to predict cardiovascular risk. In addition to models that use a combination of common laboratory parameters, *e.g.* calcium, carbon dioxide, creatinine, creatine kinase-MB, hemoglobin (Hb), glucose, mean corpuscular volume (MCV), mean corpuscular hemoglobin concentration (MCHC), platelets, potassium, red blood cell distribution width, sodium, and leukocytes or troponin I, triglycerides, urinary red blood cell count, GGT, glucose, urine specific gravity, prothrombin time, prealbumin, and urea, transcriptomic biomarkers are used today, which can predict disease with up to 96% accuracy (*46*-*48*).

The working group of the European project for the assessment of the biological age of the elderly combined laboratory parameters (*e.g.* lipid status, glucose, insulin, urate) and clinical parameters (blood pressure values, body mass index and waist-to-hip ratio). Based on the results, they concluded that oxidised low density lipoprotein (LDL), LDLox, can be considered a potential gero-biomarker indicating cardiometabolic risk as a consequence of increased metabolic stress associated with aging (*49*).

Using the deep neural network algorithm, Lee *et al.* evaluated the value of LDL-cholesterol based on the value of total cholesterol, high density lipoprotein (HDL) cholesterol and triglycerides. Compared to other methods, including the computational Fredewald method, this algorithm proved to be more accurate for the determination of LDL-cholesterol, *etc.* (*50*).

### Machine learning in laboratory hematology

The laboratory diagnosis of hematological diseases is based on the routine hematology analyzers, the traditional microscopic analysis of particular cells, and more recently on the analysis of cells using flow cytometry. Based on four parameters from the complete blood count (MCV, mean cell hemoglobin (MCH), MCHC, Hb/RBC), Azarkhish *et al.* have developed two AI-algorithms to diagnose iron deficiency anemia without determining blood iron concentration. The results showed that the AI-algorithm can estimate the iron concentration with high accuracy and precision, and that the algorithm used can contribute to the diagnosis of iron deficiency anemia (*51*). Blood analysis on automated hematologic analyzers is based on mathematical algorithms for cell classification, and changed cells are flagged. This indicates the need for morphological analysis of cells using a microscope, which requires a lot of experience of the personnel. With traditional hematological methods, hematological malignancies (leukemia and lymphoma) are often discovered when the cancer reaches an advanced stage, when there are limited prospects for successful treatment (*52*). Today, the DL methods in combination with digitalization of microscopic images makes automated image processing and cell differentiation feasible. Digitalization is achieved by the use of a computer-aided vision inspection of blood smears. Digital hematology methods, which combined digital imaging and IT, enabled the development and implementation of automated methods of digital morphological analysis of blood smears (*53*, *54*). These methods are also applied in cytology and histopathology.

Prediction and early diagnosis of hematological malignancies are crucial in improving the survival rate and reducing the mortality rate of patients. The only way to handle numerous data is to use AI. For now, ML techniques are more often applied in comparison to DL methods, because the latter are relatively newer and require larger data sets than ML. The introduction of AI-based models aims to increase the accuracy of the identification of individual cells and enable the prediction of the potential spread of a malignant disease. No standardized screening test for hematological malignancies has not yet been shown to be sufficiently reliable in the early detection of hematological malignancies. Therefore, research is underway on the effectiveness of AI in the screening, timely diagnosis and therapy of these diseases (*52*). However, this technology has limitations such as limited databases of digitized images of individual cell lines, lack of validation and standardization, systematic errors, inability to accurately classify various structural chromosomal abnormalities and bias prevent (algorithm produces results that are systemically prejudiced due to erroneous assumptions in the ML process) (*18*, *54*).

### Machine learning in allergic diseases

In recent years, ML and DL algorithms have emerged as potentially useful techniques for allergen molecule classification (*55*). These algorithms analyze enormous number of data. The key role of ML in the categorization of allergenic molecules includes: a) prediction and detection of allergenic proteins based on their sequence or structure; b) determination of the composition of amino acids, their secondary or tertiary structural components and physicochemical properties, which are very helpful in categorizing allergenic molecules; c) prediction of the cross-reactivity of similar molecules; d) identification of allergenic protein sources (plant, animal, fungal), which can contribute to the detection of food allergies; and finally, e) can help personalize allergy diagnosis as well as personalize medication implementation (*e.g.* allergen molecule specific immunotherapy).

However, the field of allergy diagnosis and treatment has also limitations, such as the need for large amounts of labeled data (specially in the case of rare allergies); there is a lack of understanding of mechanisms of many allergies; ML models may not be able to account for individual differences in patients (*56*).

Algorithms from other areas have also been developed, for example, ML algorithms have shown excellent predictive performance in the case of interpreting plasma amino acid profiles, which are determined in cases of hereditary disorders of amino acid metabolism, urea cycle and organic acidemias (*57*). The successful application of ML algorithms for the automated interpretation of steroid profiles in urine is also described (*58*).

## Artificial intelligence related obstacles

It is known that the adoption of AI technology in practice so far has been very slow, so it can be expected that the adoption of future new AI systems, regardless of their theoretical potential, will also be slow. The reason for this are the numerous obstacles that are already recognized and make it difficult to implement AI in practice. These are *e.g.*: a) new financial investments; b) privacy concerns; c) ethical aspects (patients should be informed about how their data will be used in AI research, and informed consent must be obtained); d) legal aspects (including strict rules on the collection, storage, and use of personal data); e) risks to security (cyber risk exposure – storing and processing laboratory data might cause security and privacy issues if not adequately managed), *etc.* (*59*). The most prevalent fear is that the use of AI-pods could conflict with patients’ rights, there are doubts about whether AI technology can be trusted, how rigorous AI technology evaluations are, and who evaluates AI software. Obstacles include the lack of interpretability or transparency of AI algorithms, the questionable usefulness of AI in complex multisystem diseases, doubt, since such technologies are developed mainly commercially where the main initiator is profit, which is most often reflected in the level of control, *etc*. Legal regulation of the system is certainly important. Namely, a logical question arises. Who is responsible for the results achieved through the application of AI? Are the programmers of the AI system, the health professionals who use it (so far they are responsible), the suppliers of the system, the health service that bought it, or the legal regulator that approves the AI system? The lack of understanding of the inner workings of the AI system (inadequate education) and the lack of a “gold standard” for evaluating the performance of AI algorithms is the basis of the still present mistrust in AI, which slows down the adoption of a special AI approval system (*59*).

## The future of advanced AI-supported era in clinical laboratories

Certainly, clinical laboratories will retain their fundamental role in healthcare. Moreover, thanks to advanced AI, the influence of laboratory testing on medical decision-making could be increased; the literature states that more than 70% of clinical decisions are based on laboratory results, which depends on the type of disease (*60*). The future of laboratory medicine in the era of AI will undergo significant transformations (Table 4). It can be expected that in the future, modern clinical laboratories will be directed towards the development of advanced AI-technology and that they will thus become the so-called smart laboratories (*61*, *62*). This demanding field of laboratory medicine will be included in education, both at graduate and post-graduate studies and specialization, remote monitoring and management of laboratory processes (*62*, *63*).

**Table 4:** The future of laboratory medicine in the digital era

**AI-driven progress of „smart“ laboratory**	**Example**
Automation and workflow efficiency	automated sample processing, AI-assisted quality control, workflow in real-time
Preanalytical phase	reducing inappropriate test orders, assessing of sample suitability, improving sample collection protocols
Quality control	implementation of a total quality management, reducing laboratory errors, focusing on „accuracy medicine“ and personalized medicine
Focus	progress of translational medicine and and personalized medicine
Analytical phase	consolidating conventional test, developing new tests, ensuring global standardization of tests, managing point of care testing
Postanalytical phase	verifying test interpretation algorithms, automated interpretation and reporting
Advanced image analysis	urine sediment analysis, automatically identifying cell populations by flow cytometry, digital pathology
AI-driven research and clinical trials	discovery of new biomarkers, design of more efficient clinical trials, setting international algorithmic standards
Ensuring effective diagnostic management	clinical effectiveness, output, patient safety and risk management
Communication	consultation and counseling between specialists in clinical and laboratory medicine and information technology professionals
Full integration with healthcare system	laboratory and hospital information system, integration with other hospital
AI - artificial intelligence. Adapted from references 61-63.	

Advanced AI will be successfully integrated gradually after the harmonization, validation and standardization of individual algorithms, which will be created based on the knowledge of laboratory and clinical medicine specialists and information technology experts, as well as on the basis of evidence-based laboratory medicine. A key role in the integration of advanced AI algorithms will be played by specialists in information communication technology and programmers who write code in a computer language to create the necessary software.

## Final considerations

Artificial intelligence is already integrated in clinical laboratories in numerous work domains (*e.g.* from automation and robotization of autoanalyzers, error detection, test performance, flagging of results that fall outside reference intervals, genomics and image analysis, *etc.*). It also enables easier and more precise longitudinal monitoring of biomarkers crucial for diagnosis and prediction of treatment outcomes. However, considering the potential of AI, the integration into the routine clinical laboratory is still nascent. The tools of AI continue to develop at a high speed and and offer potential improvements (testing methods, quality control, diagnostic interpretation, workflow reporting, *etc*.). So, it is to be expected that laboratories, given their specificity of obtaining a large amount of exact data, will soon be routinely using them. Artificial intelligence is based on software products, programmed algorithms that are highly dependent on IT experts. Without their knowledge and professional support, healthcare workers and laboratory experts would not be able to use these new technologies. Specialists in laboratory medicine, aware of the possibilities of AI, should acquire a basic understanding of AI technology and the algorithm development process, in order to make it easier and faster to accept upcoming AI tools. They should also participate in the development and integration of new technologies based on AI with their expertise and consulting (reciprocal relationship with IT experts and developers). First of all, a laboratory specialist should be a manager and controller of data sources, an advisor to IT specialists in his specialty/domain, and a co-developer and evaluator of algorithms. This would enable effective cooperation in planning and designing algorithms, as well as strategic evaluation and then the integration of AI technology.

Once appropriate algorithms (including biomarkers and their panels) are identified, they need to be clinically validated to be approved for routine use for specific clinical conditions, taking into account patient anthropometric characteristics as well as disease characteristics. At the same time, the methods of determining individual biomarkers must be comparable in different laboratories, that is, they must be standardized at the national and international level. Analyzers and software must be simple and reliable, and the engineers who maintain them must be available at all times.

It can be assumed that even after the harmonization, validation and standardization of individual algorithms, created on the basis of the knowledge of clinical laboratory specialists and IT experts, the integration of advanced AI technology will proceed gradually. The reason for this is numerous previously mentioned obstacles that make integration difficult, both in clinical laboratories, and in the health system in general. These are mainly obstacles of the ethical and legal type, then of the technological and social type (resistance to change), types of trust, obstacles related to the workforce, the need for more research (acquiring more clinical evidence and proven effectiveness and safety), and investment costs are not negligible either. In order to adequately solve the obstacles to integration that currently exist, strategies are recommended that include, for example, encouraging interdisciplinary collaboration (scientists, laboratory experts, clinicians, information technology experts, biostatisticians, *etc.*), defining strict algorithm validation protocols (internal and external validation with using data from multiple centers), setting international algorithmic standards, improving educational programs, continuous workforce training campaigns to follow technological progress, drafting ethical guidelines, *etc.* The primary task is to recognize, understand, define and develop appropriate approaches/strategies to overcome them and enable the integration of AI. Currently, a small number of commercially available AI models are approved for use in clinical laboratories. There is also an insufficient number of literature reviews that comprehensively addresses the research status, challenges, and future opportunities of advanced AI applications in laboratory medicine. Artificial intelligence will certainly make significant advances and changes in the work of clinical laboratories and the health system in general. The ultimate benefit is expected in providing more personalized diagnosis and treatment, but also self-control of the disease, thereby improving the quality of care and patient satisfaction. Clinical laboratories will be ready for the full and successful integration of advanced AI after establishing a balance between its potential and solving the recognized and mentioned obstacles.

## Data Availability

No data was generated during this study, so data sharing statement is not applicable to this article.
